# The fate of porcine sperm CRISP2 from the perinuclear theca before and after in vitro fertilization^†^

**DOI:** 10.1093/biolre/ioac169

**Published:** 2022-09-02

**Authors:** Min Zhang, Elizabeth G Bromfield, J Bernd Helms, Bart M Gadella

**Affiliations:** Department of Biomolecular Health Sciences, Faculty of Veterinary Medicine, Utrecht University, the Netherlands; Department of Farm Animal Health, Faculty of Veterinary Medicine, Utrecht University, Utrecht, the Netherlands; Department of Biomolecular Health Sciences, Faculty of Veterinary Medicine, Utrecht University, the Netherlands; Priority Research Centre for Reproductive Science, The University of Newcastle, New South Wales, Australia; Department of Biomolecular Health Sciences, Faculty of Veterinary Medicine, Utrecht University, the Netherlands; Department of Biomolecular Health Sciences, Faculty of Veterinary Medicine, Utrecht University, the Netherlands; Department of Farm Animal Health, Faculty of Veterinary Medicine, Utrecht University, Utrecht, the Netherlands

**Keywords:** CRISP2, porcine, sperm, perinuclear theca, decondensation, fertilization, oocyte

## Abstract

In a previous study, we reported that porcine sperm cysteine-rich secretory protein 2 (CRISP2) is localized in the post-acrosomal sheath-perinuclear theca (PT) as reduction-sensitive oligomers. In the current study, the decondensation and removal of CRISP2 was investigated during in vitro sperm capacitation, after both the induction of the acrosome reaction and in vitro fertilization. Confocal immunofluorescent imaging revealed that additional CRISP2 fluorescence appeared on the apical ridge and on the equatorial segment (EqS) of the sperm head following capacitation, likely due to cholesterol removal. After an ionophore A23187-induced acrosome reaction, CRISP2 immunofluorescence disappeared from the apical ridge and the EqS area partly not only owing to the removal of the acrosomal shroud vesicles, but to its presence in a subdomain of EqS. The fate of sperm head CRISP2 was further examined post-fertilization. In vitro matured porcine oocytes were co-incubated with boar sperm cells for 6–8 h and the zygotes were processed for CRISP2 immunofluorescent staining. Notably, decondensation of CRISP2, and thus of the sperm PT, occurred while the sperm nucleus was still fully condensed. CRISP2 was no longer detectable in fertilized oocytes in which sperm nuclear decondensation and paternal pronucleus formation were apparent. This rapid dispersal of CRISP2 in the PT is likely regulated by redox reactions for which its cysteine-rich domain is sensitive. Reduction of disulfide bridges within CRISP2 oligomers may be instrumental for PT dispersal and elimination.

## Teaser

Dispersal of the pig sperm's perinuclear the ca and complete degradation of its CRISP2 protein content was observed within 5 hours post-fertilization.

## Introduction

To achieve the capacity to fertilize an egg, mammalian spermatozoa must undergo a series of biochemical and physiological changes, defined as capacitation, in the female reproductive tract [1], or in a defined medium in vitro [2], to become competent to fertilize a mature oocyte. Sperm capacitation involves multiple signaling events including cholesterol efflux and protein tyrosine phosphorylation [3]. Eventually, these changes result in a sperm surface reordering that prime sperm to bind to the oocyte zona pellucida (ZP) and induce the subsequent zona-induced acrosomal exocytosis. Both are required for the sperm to penetrate the ZP and enable fusion with the oolemma [4]. Redistribution of lipids and proteins has been observed on the sperm surface during capacitation [5, 6]. The sperm anterior head plasma membrane is remodeled during capacitation [7, 8], and this area is considered the primary site where sperm bind to the ZP. Following capacitation, a controlled exocytotic event termed the acrosome reaction takes place, releasing acrosomal granules that likely contain enzymes believed to aid in penetrating the ZP [9]. In acrosome-reacted (AR) sperm, intra-acrosomal components become exposed at the surface of the equatorial segment (EqS) of the sperm head, which is commonly considered the first site where sperms fuse with the oolemma [10]. Numerous sperm proteins have been reported to appear at the EqS after the acrosome reaction and are essential for the sperm–egg fusion, for example, IZUMO-1 [11, 12], CD9 [13], cysteine-rich secretory protein 2 (CRISP2) [14–16].

After the sperm–egg fusion, the post-acrosomal sheath (PAS)-perinuclear theca (PT) of the sperm head is exposed to the ooplasm. The PT is a highly condensed protein structure housed in the minimal cytosolic space between the nuclear envelope and plasma membrane in the mature mammalian sperm cell. In most of the eutherian species, the PT consists of two structurally continuous regions named the sub-acrosomal layer and PAS [17]. The PAS-PT has gained attention over the past decades as it is held that the PAS-PT houses the sperm-borne, oocyte-activating factor (SOAF) responsible for oocyte activation and zygotic development [17–20]. The two favored SOAF candidates are phospholipase C zeta (PLCζ) and post-acrosomal WW domain-binding protein (PAWP). Both PLCζ and PAWP are found to be responsible for triggering Ca2^+^ oscillations within the oocyte [18–22]. Crucially, the PAS-PT is the first internal structure solubilized in the ooplasm and the sperm born cytosolic molecules are released into the oocyte cytoplasm at fertilization [17, 23]. Disulfide bond reduction has been considered as the main driving force leading to sperm head solubilization [24, 25]. This is supported by the presence of glutathione-S-transferase omega 2 (GSTO2) as an oxidative-reductive enzyme in the PAS-PT region [26]. This enzyme plays an active role in facilitating sperm nuclear decondensation likely through the reduction of disulfide bonds within sperm chromatin [27]. On the one hand, the sperm head structures including the PT must first be disassembled in the oocyte cytoplasm before sperm nuclear decondensation can take place. Before dissociation, the PT is a rigid condensed structure that protects the sperm nucleus and does not permit surrounding enzymes to initiate decondensation of the highly protamine-packed DNA. On the other hand, the proteins that are dissociating from the PT can be recruited to de- or re-stabilize sperm chromatin until male pronucleus formation [28].

The latest phylogenetic analysis shows that mammalian CRISPs have three distinct lineages, CRISP1, CRISP2, and CRISP3 [29]. CRISP4, found in some rodents, is derived from the same gene as CRISP1 [29]. CRISP1 and CRISP4 are characterized as epididymal proteins that adhere to the sperm surface during sperm epididymal maturation [30, 31]. Unlike other CRISPs, CRISP3 expression is not restricted to the reproductive tract and shows a greater diversity among species [29, 32]. Related to sperm, CRISP3 is present on ejaculated human spermatozoa, similar to human CRISP1 [33]. Interestingly, CRISP3 is found enriched in equine seminal plasma and is a valuable factor for the sperm quality [34, 35]. Downregulation of CRISP2 expression has been observed in human patients with asthenozoospermia and linked with male infertility [36–38]. CRISP2 has gained attention as it is the sole CRISP endogenously produced during spermatogenesis [39] and thus the only intracellular CRISP, while CRISP1, CRISP3, and CRISP4 are soluble proteins secreted by either the accessory sex glands or the epididymis and adsorb to the sperm surface outside the testis in the male genital tract [30, 34, 40]. Human CRISP2 reassociates with the EqS only after acrosome reaction [14] and a similar result is observed for mouse spermatozoa [16]. However, the underlying mechanisms supporting CRISP2 cellular redistribution and/or exposure is still unclear. Studies on human and rodent CRISP2 show that CRISP2 is involved in fertilization events especially at the site of the sperm–egg fusion [15, 16, 41]. Despite a morphologically normal acrosome, Crisp2^−/−^ sperms have a defect in their ability to undergo an acrosome reaction when provoked by progesterone [42]. Additionally, Crisp2^−/−^ sperms exhibit greatly lower motility due to stiffening of the midpiece of the sperm tail [42]. Thus, CRISP2 knockout males are subfertile [42, 43]. Interestingly, our previous study on porcine spermatozoa demonstrated that CRISP2 resides in the PAS-PT and that CRISP2 in the sperm head forms reduction-sensitive oligomers [44]. The domestic pig is genetically very close to humans and has often been used as a biomedical model [45]. Importantly, porcine gamete development is partly similar to human gamete development. Examples include centrosome paternal inheritance as well as timely zygotic development [10]. In this study, we have followed the fate of CRISP2 as a reporter component of the PT (i) during in vitro porcine sperm capacitation, (ii) after the calcium ionophore-induced acrosome reaction, and (iii) during in vitro fertilization (IVF) until male pronucleus formation (in the time period of 6–8 h post-co-incubation of mature porcine oocytes with porcine sperm). The dynamics of CRISP2 dispersal and degradation in the peri-fertilization period, and thus of the PT, are discussed in view of the implications for sperm chromatin decondensation and male pronucleus formation.

## Materials and methods

### Reagents and antibodies

All chemicals were obtained from Sigma (St. Louis, MO), unless otherwise stated. Goat polyclonal antibody against CRISP2 (aa77-89) (MBS422304) was obtained from MyBiosource (San Diego, CA). Mouse monoclonal antibody against phosphotyrosine (pTyr) (clone 4G10) was purchased from Sigma. Rabbit polyclonal to alpha tubulin (ab15246) was obtained from Abcam. Mouse monoclonal antibody against GAPDH was purchased from Santa Cruz Biotechnology (Santa Cruz, CA).

### Boar spermatozoa preparation

Ethical review and approval were not required for the animal study because semen samples are delivered from a commercial breeder. A written informed consent was obtained from the owners for the participation of their animals in this study. Freshly ejaculated sperm cells from highly fertile boars were obtained from a commercial breeder (Cooperative Center for Artificial Insemination in Pigs, Veghel, the Netherlands). The collected semen was diluted to 20 million sperm/mL in a commercial diluter, shipped in 80-mL sealed insemination tubes in a cool box (17°C) until use. Diluted sperm from approximately two to three different ejaculates were pooled and washed through a discontinuous Percoll (GE Healthcare) gradient (35% v/v and 70% v/v) in HEPES-buffered saline (HBS; 20 mM HEPES, 137 mM NaCl, 10 mM glucose, 2.5 mM KCl, 0.1% kanamycin, pH 7.6) for 750× *g* for 15 min, room temperature (RT). Top and interface layers were removed, and sperm pellets were further washed in HBS at 750× *g* for 10 min at RT. All solutions were iso-osmotic (290–300 mOsm/kg) and at RT before use.

### In vitro capacitation and calcium ionophore A23187-induced acrosome reaction

The capacitating medium (CM) used in this study is a modified Tyrode’s medium containing 90 mM NaCl, 10.0 mM HEPES, 3.0 mM KCl, 0.4 mM MgCl_2_, 2.0 mM CaCl_2_·2H_2_O, 0.3 mM Na_2_HPO_4_, 25 mM NaHCO_3_, 2.0 mM Na-pyruvate, 5.0 mM D-glucose, 21.6 mM sodium DL-lactate, 3.0 mg/mL bovine serum albumin (BSA) (fatty acid free), 290–300 mOsm/kg, pH 7.4. Medium supplied with neither CaCl_2_, NaHCO_3,_ nor BSA (300 mOsm/kg was achieved by compensatory NaCl addition) was defined as a non-capacitating medium (NCM). Na-pyruvate and BSA were added into the medium on the same day prior to use. Complete CM and NCM were brought to equilibrium in an incubator (38.5°C, 5% CO_2_) with loose lids or in the water bath (38.5°C) with lids on for at least 2 h, respectively, before adding to sperms. Percoll-washed sperms were suspended in the CM (1 mL, 20 × 10^6^ sperm/mL) in open vials for 2.5 h at 38.5°C in the incubator with 5% CO_2_ or incubated in NCM (1 mL, 20 × 10^6^ sperms/mL) in air-tight vials for 2.5 h at 38.5°C in a pre-warmed water bath.

Sperm cells were exposed to 5 μM calcium ionophore A23187 during the last 30 min of capacitation to induce an acrosome reaction. All sperm incubations were carried out in 5 mL polystyrene round bottom tubes (Falcon, 352,054; Life Sciences, Corning, NY). After incubation, sperm cells were spun down and washed twice with phosphate-buffered saline (PBS) (137 mM NaCl, 8.0 mM Na_2_HPO_4_, 1.5 mM KH_2_PO_4_, 2.7 mM KCl, pH 7.4) at 750× *g* for 10 min at RT. Cells were either processed for immunofluorescence or stored at −80°C for later use.

### Immunofluorescence staining of sperm

Non-capacitated (NC), capacitated (CAP), and AR sperm cells were stained using a LIVE/DEAD fixable blue kit (L23105, Thermo Scientific) to examine the cell viability following the manufacturer’s instructions. Briefly, washed sperm cells (20 × 10^6^) were resuspended in 1 mL PBS and 2 μL fluorescent-reactive LIVE/DEAD dye was added. After mixing, cells were incubated in the dark with the dye for 30 min at RT. Then the cells were washed twice with PBS at 750× *g* for 10 min to remove excessive dye and fixed in 4% paraformaldehyde (PFA) for 15 min at RT. A suspension of 100 μL sperm (10^5^ sperm/mL) was deposited in the chamber built by imaging spacers (GBL654008, Merck) on Superfrost slides (Thermo Scientific). Sperm cells were settled down for 30 min at RT and further permeabilized using 0.5% (v/v) Triton X-100 for 15 min at RT. After rising with PBS, cells were blocked using 1% (w/v) BSA in PBS for 1 h at RT, incubated overnight at 4°C with goat anti-CRISP2 or/and mouse anti-pTyr. Wells were then washed three times for 30 min in PBS before incubation for 1 h at RT with Alexa Fluor 568-conjugated donkey anti-goat IgG [H + L] or/and Alexa Fluor 488-conjugated goat anti-mouse (Thermo Scientific) and counterstaining with Alexa Fluor 488-conjugated peanut agglutinin lectin (PNA) (Thermo Scientific) or/and Hoechst 33342 (1 μg/mL Sigma) at RT for 20 min. After extensive washing with PBS, slides were mounted with FluorSave reagent (Merck Millipore) and covered with coverslips. For negative controls, primary antibodies were omitted. Observations were performed on a Leica SPE-II confocal microscope using a 63× objective (NA 1.3, HCX PLANAPO oil). The scale bar was added by Image J software (bundled with 64-bit Java 1.8.0_172, National Institutes of Health, Bethesda, MD).

### Porcine oocyte collection and in vitro maturation

Ovaries were collected from gilts from a local slaughterhouse within 2 h of slaughter. Cumulus–Oocyte complexes (COCs) were obtained by aspirating medium sized follicles (3–6 mm diameter) with an 18 g needle fixed to a vacuum pump via 50 mL conical tube and matured as previously described [46]. Briefly, COCs were recovered and washed in a modified Tyrode’s lactate-HEPES medium (TL-HEPES, 114.0 mM NaCl, 3.2 mM KCl, 2.0 mM NaHCO_3_, 0.25 mM Na-pyruvate, 0.4 mM NaH_2_PO_4_, 10.0 mM HEPES, 0.1% (w/v) polyvinylpyrrolidone (PVP), 10.0 mM Na-lactate, 0.5 mM MgCl_2_·6H_2_O, 2.0 mM CaCl_2_·2H_2_O). COCs were further washed twice in pre-warmed Medium 199 (Gibco) supplemented with 2.2 mg/mL NaHCO_3_ (Medium C incomplete) and washed once in pre-equilibrated Medium C incomplete supplemented with 10% porcine serum, 1 mM Na-pyruvate, and 0.6 mM cysteine (oocyte maturation medium; OMM). COCs were transferred in groups of 40–50 per well in to a four-well dish containing 500 μL equilibrated OMM-I (OMM supplemented with 0.2 mM cysteamine, 0.05 IU/mL recombinant human follicle-stimulating hormone (rhFSH, Organon, Oss, The Netherlands), 100 units/mL penicillin, and 100 μg/mL streptomycin (Gibco)) and incubated at 38.5°C with 5% CO_2_ for 22 h. After 22 h, COCs were then washed twice in OMM-ІІ (OMM-I without rhFSH) and then placed in 500 μL OMM-II for an additional 20 h to reach the metaphase II stage.

### In vitro fertilization

The expanded cumulus cells were removed by using a micropipette set at 150 μL and pipetting the contents of the dish in and out 30 times. Denuded oocytes were then washed twice in equilibrated IVF medium (113.1 mM NaCl, 3.0 mM KCl, 20.0 mM Tris, 11.0 mM glucose, 1.0 mM caffeine, 7.5 mM CaCl_2_·2H_2_O, 0.1% BSA (fatty acid free), 5 mM Na-pyruvate (add on the day of fertilization), 100 units/mL penicillin, and 100 μg/mL streptomycin, 275–290 mOsm/kg, equilibrated overnight in the incubator at 38.5°C in 5% CO_2_) and transferred in groups of 40–50 oocytes into a four-well dish containing a 500 μL IVF medium and cultured at 38.5°C with 5% CO_2_ until adding sperms. Freshly ejaculated sperm cells (from three different boars) were mixed and washed twice in an IVF medium at 700× *g* for 4 min at RT. Sperm pellets were resuspended in 3 mL of the IVF medium and pre-stained with 0.5 μM MitoTracker Red (Thermo Scientific) in 37°C water bath for 15 min. After twice washing with IVF medium, sperm suspensions were added into oocytes at a final concentration of 2.5 × 10^5^ sperm/mL and co-incubated at 38.5°C with 5% CO_2_ for 6–8 or 24 h. At 24 h post-IVF, the presumptive zygotes were removed from the IVF wells, washed twice in pre-equilibrated synthetic oviductal fluid (SOF) medium [47] and placed in groups of 40–50 per 500 μL SOF medium for further development at 38.5°C with 5% CO_2_ and 7% O_2_. The cleavage rate (≥2-cell) and blastocyst formation of embryos were assessed 48 h and 7 days after IVF, respectively (Table 1).

**Table 1:** The cleavage (≥2-cell) and blastocyst formation

No. of examined oocytes	≥ 2-Cell (at 48 h)	Blastocysts (at day 7)
Group 45	27	3
47	19	3
50	41	3
50	40	7
46	25	7
39	27	2
	≥ 2-Cell rate (%)	Blastocysts relative to total oocytes (%)
Total 277	64.3 ± 16.0%	8.9 ± 4.5%

Six groups from three independent experiments. The cleavage (≥2-cell) and blastocyst formation of embryos were assessed 48 h and 7 days after IVF, respectively. The number of 2-cell and blastocyst stages were observed per group. The percentages for the cleavage (≥2-cell) and blastocyst rates are expressed as the mean ± standard deviation (SD).

### Immunostaining of oocytes/zygotes

Immunostaining analysis of oocytes was carried out as previously described [20] with minor modifications. Before fixation, the ZP was removed by briefly incubating with 0.5% (w/v) protease in TL-HEPES. The zona-free zygotes were fixed in 4% PFA for 15 min at RT and stored in 0.1 M phosphate buffer (PB)-0.01% (w/v) PVP at 4°C, if not used immediately (no longer than 5 days). Oocytes were permeabilized in 0.1 M PB-0.01% (w/v) PVP with 0.1% Triton X-100 for 1 h at RT, then blocked in 0.1 M PB-0.01% PVP (w/v) with 1% (w/v) BSA for 45 min at RT. After this, zygotes were incubated with a CRISP2 antibody (1:100) diluted in 0.1-M PB-0.01% PVP (w/v) with 1% (w/v) BSA at 4°C, overnight. After three times washing in 0.1 M PB-0.01% PVP (w/v), oocytes were incubated with Alexa Fluor 488-conjugated or Alexa Fluor 568-conjugated donkey anti-goat IgG [H + L] (Thermo Scientific) for 1 h at RT in the dark. After extensive washing, oocytes were counterstained with 20 μg/mL Hoechst 33342 for 10 min at RT. Oocytes were mounted in imaging spacers (GBL654008, Merck) on Superfrost slides (Thermo Scientific) with FluorSave reagent (Merck Millipore) and covered with coverslips. Observations were performed on a Leica SPE-II confocal microscope using a 63× objective (NA 1.3, HCX PLANAPO oil).

**Figure 1:**
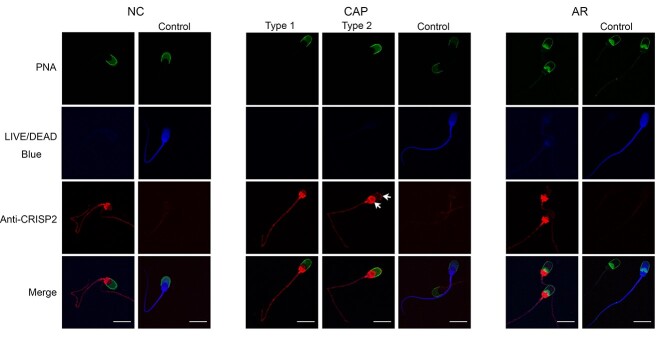
The distribution of porcine sperm CRISP2 during in vitro capacitation and the acrosome reaction. After incubation, NC, CAP, and AR sperm cells were washed and labelled with a live/dead blue dye before fixation and permeabilization. Sperm cells were then labelled with anti-CRISP2 and Alexa Fluor 568-conjugated secondary antibodies (red) followed by Alexa Fluor 488-conjugated PNA (green). The CRISP2 primary antibody was omitted and representative dead cells with bright blue signals were shown in control. Additional signals were observed on the apical ridge of sperm head and the EqS (arrows). Three ejaculates from different boars were mixed as one biological replicate and this experiment was replicated three times. Scale bar = 10 μm.

### Sodium dodecyl sulfate-polyacrylamide gel electrophoresis (SDS-PAGE) and immunoblotting

An equal number of sperm cells were lysed in the same volume of RIPA lysis buffer (Thermo Scientific) with freshly added protease inhibitors containing aprotinin, leupeptin, pepstatin, and phenylmethylsulfonyl fluoride (PMSF) (Thermo Scientific) on ice for 30 min with mixing. Cell debris was removed by centrifugation at 14000 *g* for 15 min, at 4°C. Supernatants were transferred to new tubes and denatured in a 4× SDS sample buffer (200 mM Tris–HCl, pH 6.8, 10% β-mercapto-ethanol, 8% SDS, 0.08% bromophenol blue, 40% glycerol) and boiled for 10 min. The same amount of each lysate was loaded on to an SDS-PAGE gel (5% stacking gel, 12% resolving gel) and blotted onto 0.2-μm nitrocellulose membranes (GE Healthcare, Piscataway, NJ) at 100 V for 1 h. After blocking for 3 h at RT in 5% (w/v) BSA in PBS with 0.05% (v/v) Tween-20 (PBST), membranes were incubated with primary antibodies (diluted in PBST with 1% BSA) overnight at 4°C. After three washes in PBST for 15 min, membranes were incubated with horse radish peroxidase (HRP)-conjugated secondary antibodies (mouse anti-goat HRP IgG, sz-2354, Santa Cruz, CA; goat anti-rabbit and mouse HRP IgG, P0448, Agilent) for 1 h at RT. After rinsing four times in PBST for 20 min, membranes were developed using chemiluminescence (ECL-detection kit; Supersignal West Pico, Pierce, Rockford IL). The protein band size was determined using a PageRuler Plus pre-stained protein ladder, 10–250 kDa (Thermo Scientific).

## Results

### Exposure of CRISP2 on the apical ridge and the EqS of the sperm head during capacitation

**Figure 2:**
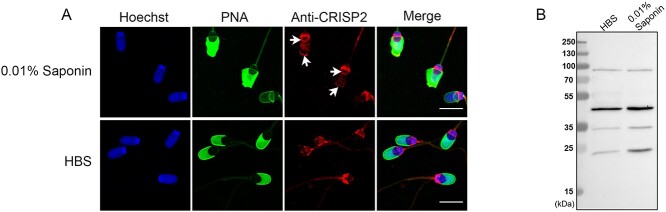
Saponin incubation with ejaculated spermatozoa mimics the exposure of CRISP2 at the apical ridge and the EqS of capacitated sperm. Percoll washed sperm cells were incubated in 0.01% Saponin supplied in HBS for 10 min at 37.5°C. After incubation, sperm cells were washed and sampled for immunostaining and immunoblotting analysis. (A) Sperm cells were fixed and permeabilized, probed with anti-CRISP2 followed by Alexa Fluor 568-conjugated (red) secondary antibodies, counterstained with Alexa Fluor 488-conjugated PNA (green) and Hoechst 33342 (blue). Scale bar = 10 μm. (B) Immunoblotting analysis of CRISP2 on the extracts from Saponin treated sperm probed with anti-CRISP2.

We have characterized the biochemical properties of CRISP2 in ejaculated boar spermatozoa in earlier work [44]. Here we explored the fate of CRISP2 during in vitro capacitation and during the calcium ionophore-induced acrosome reaction. To perform this study, we first focused on immunostaining of CRISP2 in NC, CAP, and AR porcine sperm. This was performed in the presence of PNA to assess the acrosome status. The results demonstrated that the fluorescent signals of CRISP2 in NC sperms were bright in the post-acrosomal region and the connecting piece, with weak signals in the sperm tail, as previously reported [44] (Figure 1, Supplementary Figure S1A). Interestingly, after incubation in the CM, a population (34.2 ± 3.8%; Table 2) of sperms with intact acrosomes showed strong CRISP2 labeling at the EqS region and the apical ridge of the sperm head (labeling type 2), and this CRISP2 labeling pattern was absent in NC sperms (Figure 1, Supplementary Figure S1B). While the majority possessed a staining pattern (labeling type1) that was consistent with NC sperms (Figure 1, Supplementary Figure S1B). To eliminate the possibility that staining pattern in type 2 was specific associated with cell death, a live/dead blue staining was used to assess sperm viability and revealed that type 2 stained sperm cells had no preference to deteriorated versus intact sperm cells. After the calcium ionophore-induced acrosome reaction, CRISP2 signals at the EqS and the apical ridge were not visible, but CRISP2 labeling was detected at the subdomain of EqS (EqSS) (Figure 1, Supplementary Figure S1A). To address the point that additional CRISP2 staining of capacitated sperms was likely an exposure, fresh spermatozoa was incubated with 0.01% saponin. Interestingly, the staining pattern of CRISP2 at the apical ridge and the EqS of the sperm head was mimicked. CRISP2 signal was present in the apical ridge when spermatozoa possessed a PNA-positive and swollen acrosome (Figure 2A). Further, immunoblotting analysis of CRISP2 showed that Saponin treatment did not cause visible loss of CRISP2 from sperm cells (Figure 2B).

**Table 2:** Type 2 labeling of CRISP2 in pig sperm after *in vitro* capacitation

No. of examined sperms	Sperms showing type 2 labeling
Group 206	77
230	81
217	65
	(%) Sperm showing type 2 labeling
Total 653	34.2 ± 3.8%

In the case of *in vitro* capacitated sperm, the exposure of CRISP2 on the apical ridge and the EqS (labeling type 2) was recorded. This experiment was replicated three times and the number of sperms examined as well as the number of those sperms with type 2 labeling for CRISP2 were expressed for each group. The percentage of sperms with type 2 labeling is expressed as the mean ± SD.

### Additional appearance of CRISP2 is specific for capacitated sperm cells that show protein tyrosine phosphorylation

**Figure 3:**
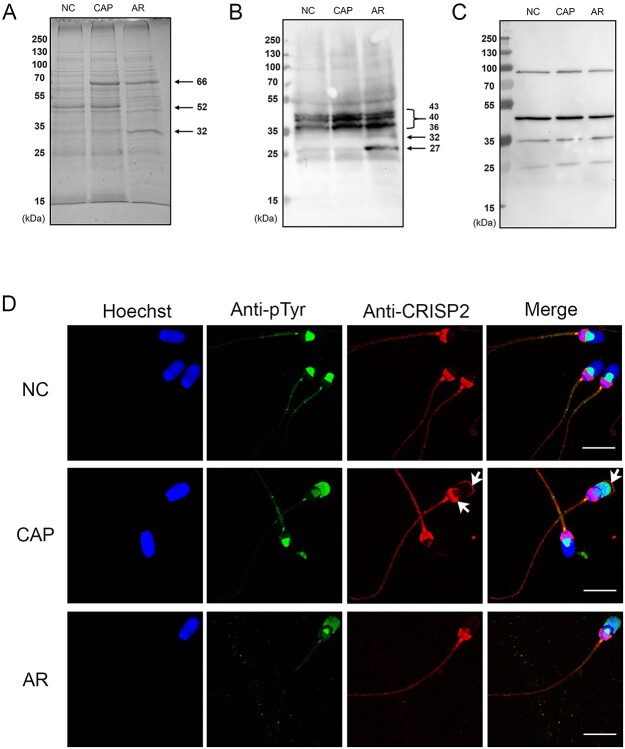
Exposure of CRISP2 at the apical ridge and the EqS of the sperm head during capacitation is an event associated with tyrosine phosphorylation. (A) Lysates from NC, CAP, and AR sperm cells were analyzed by SDS-PAGE and Coomassie blue staining. Immunoblotting analysis of tyrosine phosphorylation and CRISP2 on the extracts from NC, CAP, and AR sperm cells probed with anti-pTyr (B) and anti-CRISP2 (C) antibodies. (D) NC, CAP, and AR sperm cells were co-incubated with anti-CRISP2 and anti-pTyr, labelled with Alexa Fluor 568-conjugated (red) and Alexa Fluor 488-conjugated second antibodies (green) and counterstained with Hoechst 33342 (blue). This experiment was replicated three times. Scale bar = 10 μm.

To confirm that the translocation of CRISP2 to the EqS and apical region was dependent on capacitation, protein tyrosine phosphorylation was analyzed to confirm the capacitation status of the cells. Coomassie brilliant blue staining analysis of lysates from NC, CAP, and AR sperm was conducted to view total proteins loading and protein changes. As indicated in Figure 3A, the total protein amount among the conditions was comparable, with three protein bands that changed depending on capacitation and acrosome reaction treatments: (i) an ~ 66-kDa protein band was absent in NC spermw and present in CAP and AR sperm. This likely corresponds to BSA, which was not present in the NCM. (ii) An ~ 52-kDa protein band was present in NC and CAP sperms, but greatly decreased in AR sperms. This protein band likely represents proacrosin. Finally, (iii) the ~32-kDa protein band associated with AR sperm is in line with the corresponding increased tyrosine phosphorylated protein detected by immunoblots in Figure 3B. Immunoblotting analysis on lysates from NC, CAP, and AR sperms showed that the intensity of ~43, ~40, and ~36 kDa protein tyrosine-phosphorylation bands was increased when sperm were incubated in CM. Additional bands of ~32 and ~27 kDa also appeared after calcium ionophore-induced acrosomal exocytosis (Figure 3B). Previous studies have reported that protein tyrosine phosphorylation is enhanced at the acrosome in capacitated boar sperms, with consistently signals being present in the EqSS [48]. Similar results were observed in our study. In NC sperms, protein tyrosine phosphorylation was restricted to the EqSS (Figure 3D). After capacitation, a representative 25% sperms showed the tyrosine phosphorylation signal spreading over the acrosome. Among those, a representative 76% sperms showed additional CRISP2 labeling at the apical ridge and EqS (Figure 3D). After induction of the acrosome reaction, additional labeling of CRISP2 in the apical ridge was not observed despite protein tyrosine phosphorylation fluorescence labeling the acrosome region. Moreover, CRISP2 was colocalized with tyrosine phosphorylation fluorescence in the EqSS in AR sperms (Figure 3D). Immunoblot analysis of CRISP2 on lysates from NC, CAP, and AR sperm cells was shown in Figure 3C.

### Distribution of CRISP2 of sperm bound to ZP

**Figure 4:**
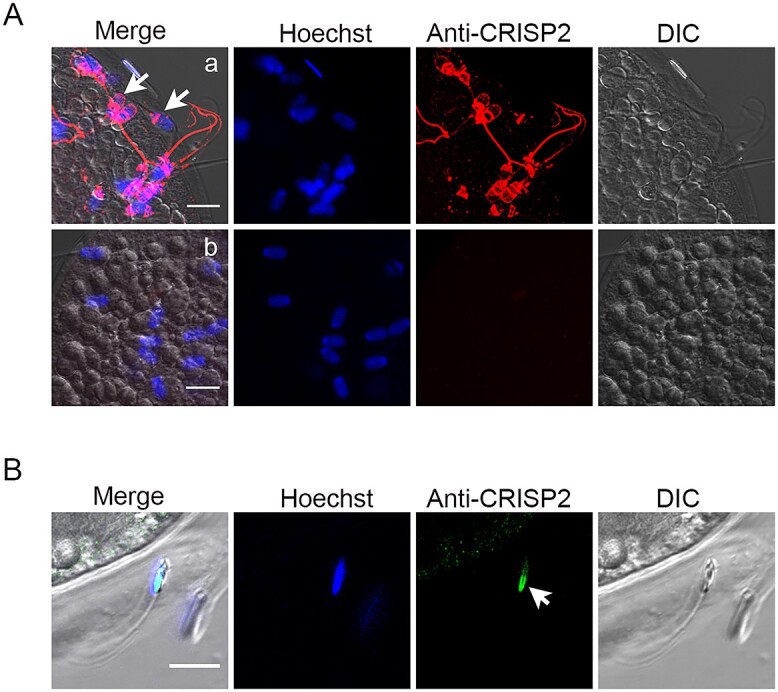
The distribution of sperm CRISP2 in zona-bound and zona-penetrated spermatozoa. Porcine oocytes and sperm were co-incubated for 6–8 h after insemination. Zygotes were fixed and permeabilized for immunochemistry analysis. (A) Zygotes were labelled with anti-CRISP2 (a) followed by Alexa Fluor 568-conjugated (red) secondary antibodies and counterstained with Hoechst 33342 (blue). (b) The anti-CRISP2 was omitted. (B) Immunolocalization of CRISP2 of sperm penetrated in zona detected by Alexa Fluor 488-conjugated (green) secondary antibodies. Scale bar = 10 μm.

To further explore how CRISP2 is distributed post-fertilization, IVF was performed on matured porcine oocytes by coincubation with porcine sperms. Initial experiments focused on investigating the distribution of CRISP2 in the sperms that were bound to the oocyte ZP. Immunostaining of CRISP2 revealed CRISP2 signal at the apical ridge, the EqS, the post-acrosomal region of the sperm head and tail (Figure 4Aa). This distribution of CRISP2 was consistent with what we described above for CAP sperm that showed increased protein tyrosine phosphorylation. Sperms that were penetrating the ZP lost the signal on the apical ridge as well as on the EqS, but the immunofluorescence of CRISP2 in the post-acrosomal region was retained (Figure 4Aa). In Figure 4B, another capture of sperm penetrating the ZP showed that CRISP2 stayed condensed in the post-acrosomal region. A recent study has shown that there is no CRISP2 mRNA expression in porcine ovary [49]. In our study, mature oocytes were collected and processed for immunoblotting analysis. Ejaculated sperm cells were used as a positive control. We have described in our earlier study that 8 M urea treatments cause high molecular CRISP2 complexes disassociation into a monomer [44]. Here, denuded oocytes were also solubilized in 8 M urea to acquire an efficient denature of oocyte proteins. Our result revealed that no CRISP2 signal was detected on the immunoblot of the porcine oocytes lysates (Supplementary Figure S2). Thus, the CRISP2 signal detected in Figure 4A/B was definitely coming from CRISP2 within boar spermatozoa.

### Rapid dispersal of sperm CRISP2 prior to sperm nuclear decondensation

**Figure 5:**
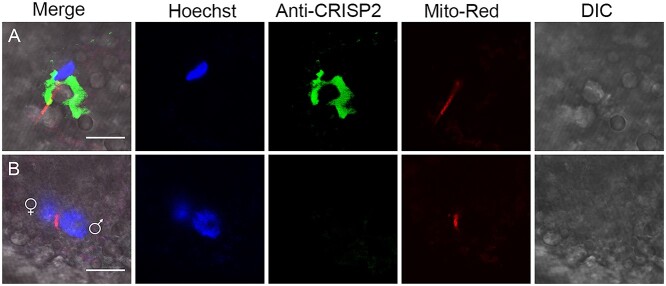
The fate of porcine sperm CRISP2 after incorporation with oocyte cytoplasm. Porcine oocytes and MitoTracker Red pre-stained sperm were co-incubated for 6–8 h after insemination. Zygotes were stripped of ZP using 0.5% (w/v) protease prior to fixation and permeabilization, labelled with anti-CRISP2 detected by Alexa Fluor 488-conjugated (green) secondary antibodies and counterstained with Hoechst 33342 (blue). (A) Rapid dispersal of sperm CRISP2 was observed in the zygote after incorporation while the sperm nucleus was still condensed. Moreover, the mitochondria were compacted. (B) CRISP2 signals were beyond detection in the two-pronuclear stage. Scale bar = 10 μm.

Intrigued by the findings that CRISP2 is a component of the PAS-PT and its important roles in early events of fertilization, we sought to investigate the fate of sperm CRISP2 in fertilized pig oocytes. Oocytes were co-incubated with fresh boar spermatozoa preloaded with fluorescent MitoTracker Red probe, cultured and sampled for CRISP2 detection. The dissociation of sperm CRISP2 from the PAS-PT was observed within the oolemma, while the sperm nucleus was still fully condensed and the entire mitochondrial sheath was still intact (Figure 5A). Remarkably, sperm CRISP2 was undetectable in zygotes that were in the two-pronuclear stage, while sperm mitochondria were still linear arranged (Figure 5B, Supplementary Figure S3B). Multilayer scanning (*z*-stack, step size 1 μm) was recorded to show the CRISP2 and sperm mitochondrial sheath originating from the fertilizing sperm cell (Supplementary Files 1 and 2). Another fertilizing sperm was imaged and showed CRISP2 dispersal as well as a decreased intensity of CRISP2 immunofluorescence, while the sperm nucleus was still condensed (Supplementary Figure S3A). This indicates that the PT dissociation (as imaged with CRISP2 immunolabeling) and degradation events occur earlier than sperm chromatin decondensation and degradation of the sperm mitochondria.

## Discussion

In the past few decades, CRISPs have been extensively investigated due to their proposed functions in reproductive events. Crucially, triple and quadruple CRISP knockout models have been described to have severely subfertile phenotypes and generate a higher number of sterile males [50], indicating that the CRISP family are indispensable for male fertility. The outcome that single and double knockout mice males are still fertile under various laboratory conditions is explained by compensatory mechanisms within homologous family proteins [42, 50–53]. However, the intracellular sperm CRISP2 cannot be compensated by other CRISPs that are associated with the sperm surface. CRISP2 is crucial for flexible sperm midpiece development, suggesting that CRISP2 holds independent functions contributing to fertility process [42, 50].

In the present study, we investigated the fate and organization of boar sperm CRISP2 during in vitro capacitation, calcium ionophore-induced acrosome reaction and throughout the first 6–8 h of IVF of porcine oocytes. Our data demonstrated that following capacitation, additional CRISP2 immunofluorescence was detected on the apical ridge as well as the EqS of the sperm head. Interestingly, this staining pattern is similar to that we have obtained after incubating ejaculated porcine sperm with 0.01% saponin. Saponin is a mild detergent and can be used at very low concentrations to selectively permeabilize the plasma membrane but no other interior membrane compartments [54]. Saponin removes cholesterol from membranes with little disintegration of the membranes themselves [54]. Physiologically, cholesterol removal is also observed in capacitating sperm cells [8, 55, 56]. Moreover, we showed previously that additional CRISP2 labeling of the EqS on ejaculated spermatozoa is observed only after sonication, not in untreated, intact sperms [44]. A similar phenomenon has been reported for equatorin, a sperm head equatorial protein, which is exposed at the EqS after sonication and protease inhibitors are unable to prevent the exposure [57]. A proteolytic reaction is not likely involved during the exposure of CRISP2 after sonication as the sonication medium was enriched with PMSF in order to inhibit proteases. Physiologically, the exposure of CRISP2 on the apical ridge and the EqS was only associated with capacitated sperms, as indicated by positive tyrosine phosphorylation labeling over the acrosome [48]. Protein tyrosine phosphorylation is a capacitation marker with the process thought to occur simultaneously with cholesterol depletion [8]. Sperm membrane cholesterol efflux is part of a signal transduction pathway involving elevated cAMP levels and protein kinase A activity leading to protein tyrosine phosphorylation [3, 58]. Evidence demonstrates that cholesterol restricts the freedom of membrane proteins to undergo conformational changes [59]. The fact that human CRISP2 binds sterols in vitro may suggest a sterol binding function of CRISPs in sperm maturation and fertilization process [60]. Additionally, studies show that cholesterol removal disassociates the interaction between the proteins and caveolin, leading to the activation of caveolin-interacting proteins [61, 62]. Thus, we conclude that the exposure of CRISP2 on the apical ridge and the EqS is a capacitation-associated phenomenon likely driven by cholesterol efflux. Given that the capacitation status of sperms is positively correlated to sperm–zona binding and acrosome reaction [63] as well as that the apical ridge of sperm head membrane has high affinity for sperm–zona binding after capacitation [64–66], our data suggest that CRISP2 on the apical ridge and the EqS of capacitated cells may be indicative of those cells that can bind to the ZP. This idea was supported by the immunofluorescent staining of CRISP2 of zona-bound sperm that CRISP2 was located on the apical ridge, the EqS, the post-acrosomal region, and the sperm tail.

It has been reported that the EqS still holds intact membranes even after the acrosome reaction [67]. Unexpectedly, our results revealed that the CRISP2 signal on the EqS disappeared after the acrosome reaction, whereas CRISP2 immunofluorescence was detected in a EqSS. Initially, the EqSS was characterized as an unusual semicircular substructure within the EqS by atomic force microscopy in Artiodactyla spermatozoa [68]. The EqSS develops during sperm epididymal maturation [68] and is enriched in tyrosine phosphorylated proteins such as sperm acrosome-associated protein 1 and heat-shock protein 70 [69, 70]. The presence of CRISP2 in the EqSS was confirmed in our previous study when sonicated sperm heads are extracted in 0.2% Triton X-100 and this signal disappears after 0.1 M NaOH extraction as the PAS-PT residing CRISP2 [44]. It indicates that CRISP2 in the EqSS underlying the PT shares similar biochemical extractability properties of CRISP2 in the PT itself. Topographical changes in the EqSS have been observed when sperm are introduced to calcium ionophore A23187, and thus, this area has been speculated to initiate the sperm–egg fusion within Artiodactyla [68]. However, no prominent structural changes or modifications of PT following the acrosome reaction have yet been reported. The loss of CRISP2 immunofluorescence from the EqS after ionophore A23187-induced acrosome reaction suggests that CRISP2 is not directly involved in sperm–egg fusion at this site.

Our key findings of this manuscript suggest that the PAS-PT residing CRISP2 in boar spermatozoa [44] is dispersed rapidly in the oocyte cytoplasm, which may be needed for subsequent nuclear decondensation and male pronucleus formation. In an attempt to quantify CRISP2 decondensation and its subsequent degradation, we have recorded 28 fertilized pig oocytes 6–8 h post-fertilization. Four of them showed the two pronuclear stages in which CRISP2 labeling was not visible (as shown in Figure 5B and Supplementary Figure S3B). The other 24 of them showed a still condensed sperm nucleus and clear MitoTracker Red staining. However, only 5 out of these 24 fertilized oocytes showed CRISP2 dispersal, whereas the other 19 had already degraded their CRISP2. This indicates that CRISP2 dispersal and degradation occur rapidly at fertilization and much earlier than sperm chromatin decondensation. The rapid dispersal of CRISP2 is likely due to the disassembly of disulfide bonds facilitated by GSTO2 [24, 27]. In the sperm head, CRISP2 resides in the condensed PT structure and is involved in formation of reduction-sensitive oligomers at its cysteine-rich domain, which fits with the conversed amyloidogenic behavior of CAP proteins [44, 71]. The removal of PAS-PT from sperm head is indispensable for sperm chromatin decondensation and the released molecules from PAS-PT are essential for oocyte activation [17]. The compacted organization of CRISP2 in the PAS-PT and its rapid disassociation from the sperm nuclear envelope post-fertilization (more or less immediately when the PAS-PT is exposed to oocyte cytoplasm) fits the model proposed by Sutovsky and Oko [17]. Notably, CRISP2 dispersal is complete prior to any signs of male nuclear decondensation, raising the possibility that disulfide bond reduction is the way to initiate PAS-PT solubilization. The oxidation of free thiol groups in proteins is reversibly regulated by redox reactions and is crucial in many cellular functions [71, 72]. Sperm cells carry physiological levels of reactive oxygen species that play an important role in sperm maturation, capacitation, acrosome reaction, and sperm–egg fusion [73]. We speculate that CRISP2 is modified concomitantly with PT condensation in the elongating phase of spermatids, thus serving as a scaffold stabilizing the rigidity of PT as well as hampering the activity of certain proteins. Soon after the sperm–egg incorporation, reduction of disulfide bridges within CRISP2 as a transduction signal leads to the solubilization of PT contents into the oocyte cytoplasm and subsequently the sperm nuclear decondensation. Once inside the oocyte, the decondensing PT will liberate other PT components into the oocyte’s cytosol that may be relevant for post-fertilization changes in the oocyte. Of specific interest are the CRISP2-interacting PT proteins: PAWP and Ras-related protein Rab-2B (RAB2B) [74]. The release of RAB2B, can be important for locally regulating vesicle transport and membrane fusion [75]. Sperm that interacted with the oolemma showed condensed RAB2B staining (unpublished observations) supporting the idea that this PT component is introduced into the oocyte’s cytosol at fertilization. A similar release of PAWP and phospholipase C ζ from the PT into the oocyte’s cytosol may be involved in the induction of Ca^2+^ oscillations required for activating the oocyte [19, 20]. Finally, it is notable that CRISP2 is degraded soon after PT dispersal and is eliminated before a male pronucleus is formed. The PT structure is enriched in proteasome subunits (in fact it contains all subunits for re-assembly of a functional proteasome) [74]. The group of Sutovsky has designed a cell free system to follow the dispersal and breakdown of sperm structures [76]. Such an approach in combination with redox titrations on sperm heads can be used to follow PT dispersion and break down in vitro. In this way, it is possible to demonstrate that the reassembly of these proteasome subunits into functional proteasomes will facilitate the destruction of the PT structure and other accessory sperm components, as shown here for CRISP2.

## Author contributions

MZ conducted the experiments, contributed to figure preparation, editing, and wrote the draft of the manuscript. EGB and JH provided a critical appraisal of the data and reviewed the manuscript. BMG conceived the study and contributed to supervision, manuscript revision, and editing.

## Supplementary Material

Supplementary_materials_ioac169

## Data Availability

The data underlying this article will be shared on reasonable request to the corresponding author.
