# Supplementary material for: The fate of porcine sperm CRISP2 from the perinuclear theca before and after in vitro fertilization
Source: Biol Reprod. 2022 Sep 2;107(5):1242–53. doi: 10.1093/biolre/ioac169 (PMC9663942; doi:10.1093/biolre/ioac169)
Supplement: Supplementary_materials_ioac169 [file supplementary_materials_ioac169.docx]

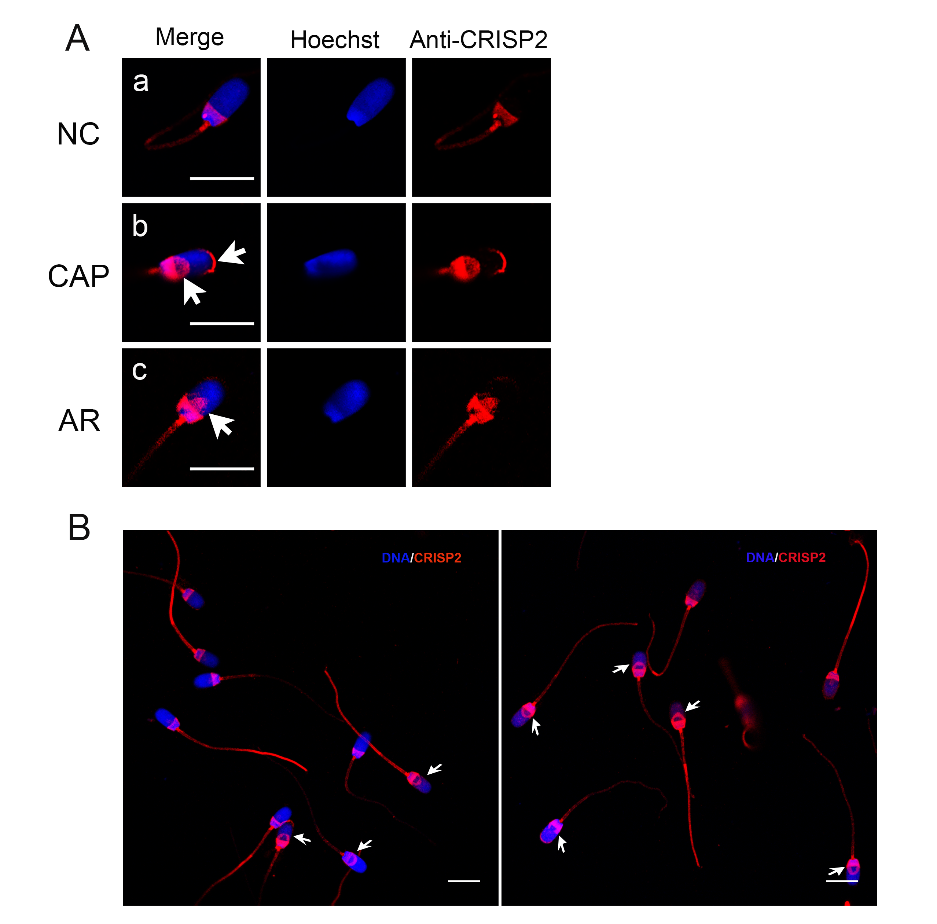


**Supplementary figure S1. The distribution of CRISP2 following in vitro capacitation and acrosome reaction.** (A) Representative single cell images showed the distribution of CRISP2 following capacitation and acrosome reaction. (a) CRISP2 was present in the post-acrosomal region, the connecting piece and faint signal in the sperm tail of NC sperm. (b) Additional signals of CRISP2 were detected on the apical ridge and the EqS of CAP sperm. (c) CRISP2 immunofluorescence was revealed in the subdomain of EqS (EqSS) of AR sperm. (B) In the case of capacitated sperm, overview on several cells were included to show the exposure of CRISP2 on the apical ridge and the EqS of the sperm head (arrowed). 2~3 ejaculates from different boars were mixed as one biological replicate and this experiment was replicated three times. Scale bar = 10 μm.


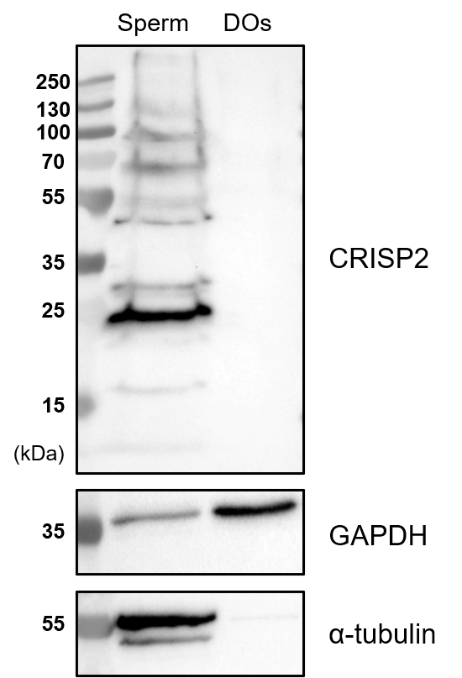


**Supplementary figure S2. CRISP2 is not present in the porcine oocyte.**

After in vitro maturation, porcine oocytes were denuded by removing cumulus cells. Ejaculated sperm cells and denuded oocytes were lysed in 8 M urea as we described before [44]. 5 x10^5^ sperm cells per lane, 20~30 oocytes per lane. Immunoblotting analysis of CRISP2 on the lysates from sperm and oocytes probed with anti-CRISP2. Membrane was stripped and re-probed with anti-GAPDH and α-tubulin.


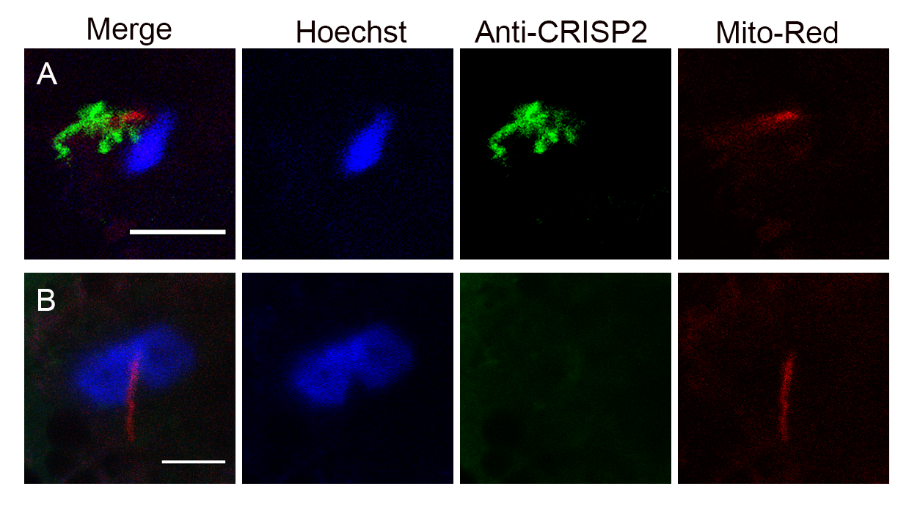


**Supplementary figure S3.** (A) Another fertilizing sperm was imaged and showed CRISP2 dispersal, while the sperm nucleus was still condensed. (B) An image of zygotes showed that sperm CRISP2 was undetectable in zygotes that were in the two-pronuclear stage, while sperm mitochondria were still linear arranged. Scale bar = 10 μm.

**Supplementary file 1&2. Multilayer scanning (z-stack, step size 1 μm) of the zygotes in Figure 5A and 5B, respectively.**

Supplementary file 1

Supplementary file 2
